# P-853. Assessment of Pharmacist Led Follow-up of Microbiology Results Post Hospitalization

**DOI:** 10.1093/ofid/ofaf695.1061

**Published:** 2026-01-11

**Authors:** Punit J Shah, Brittany Yalamanchili, Sarfraz Aly

**Affiliations:** Houston Methodist Sugar Land Hospital, Sugar Land, TX; Houston Methodist Sugar Land Hospital, Sugar Land, TX; Houston Methodist Sugar Land Hospital, Sugar Land, TX

## Abstract

**Background:**

Significant medical errors have been described during transitions of care (TOC) from inpatient to outpatient health care settings. A common gap in TOC from acute hospitalization is lack of follow up on pending inpatient microbiology cultures. Although emergency department culture call-back programs have been frequently described, published literature from inpatient settings is limited.

**Methods:**

This was a descriptive, single-center retrospective study conducted in a 356-bed community hospital. All microbiology cultures that resulted after a patient’s inpatient hospitalization were reviewed by a clinical pharmacist. The study period was from November 1, 2024 to March 31, 2025. A real-time clinical decision support tool was utilized to generate alerts when a microbiology culture resulted positive with the growth of a microorganism after the patient’s inpatient discharge. A clinical pharmacist evaluated these alerts and if warranted, contacted the physician for modification of antimicrobial therapy. The pharmacist would send prescriptions to the patient’s outpatient pharmacy and provide patient education as clinically indicated. The primary outcome was the percentage of patients requiring a pharmacist intervention, and the secondary outcome was 30-day all cause readmission rates.

**Results:**

During the study period, 496 unique patients with 1,088 alerts of microbiology cultures were identified and reviewed. Clinical pharmacists intervened on 87 patients (18%), of which 16 (18.4%) interventions were assigned a category 1 severity rating (failure to intervene could result in significant patient harm). Thirty-six (41.4%) interventions were assigned a category 2 severity rating (failure to intervene could result in minor or temporary patient harm), and 35 (40.2%) interventions were assigned a category 3 severity rating (interventions included therapy optimization). Acute bacterial skin and skin structure infections and osteomyelitis made up the majority 47% (n=41) of the interventions. Of the patients intervened on by a pharmacist, the 30-day all cause readmission rate was 10% (9/87).

**Conclusion:**

Post-hospitalization review of microbiology results can improve patient safety during TOC, and provides an avenue to further improve patient care and health system outcomes.

**Disclosures:**

All Authors: No reported disclosures

